# Brain Signature of Chronic Orofacial Pain: A Systematic Review and Meta-Analysis on Neuroimaging Research of Trigeminal Neuropathic Pain and Temporomandibular Joint Disorders

**DOI:** 10.1371/journal.pone.0094300

**Published:** 2014-04-23

**Authors:** Chia-shu Lin

**Affiliations:** Department of Dentistry, School of Dentistry, National Yang-Ming University, Taipei, Taiwan (ROC); Boston Children's Hospital and Harvard Medical School, United States of America

## Abstract

Brain neuroimaging has been widely used to investigate the bran signature of chronic orofacial pain, including trigeminal neuropathic pain (TNP) and pain related to temporomandibular joint disorders (TMD). We here systematically reviewed the neuroimaging literature regarding the functional and structural changes in the brain of TNP and TMD pain patients, using a computerized search of journal articles via PubMed. Ten TNP studies and 14 TMD studies were reviewed. Study quality and risk of bias were assessed based on the criteria of patient selection, the history of medication, the use of standardized pain/psychological assessments, and the model and statistics of imaging analyses. Qualitative meta-analysis was performed by examining the brain regions which showed significant changes in either brain functions (including the blood-oxygen-level dependent signal, cerebral blood flow and the magnetic resonance spectroscopy signal) or brain structure (including gray matter and white matter anatomy). We hypothesized that the neuroimaging findings would display a common pattern as well as distinct patterns of brain signature in the disorders. This major hypothesis was supported by the following findings: (1) TNP and TMD patients showed consistent functional/structural changes in the thalamus and the primary somatosensory cortex, indicating the thalamocortical pathway as the major site of plasticity. (2) The TNP patients showed more alterations at the thalamocortical pathway, and the two disorders showed distinct patterns of thalamic and insular connectivity. Additionally, functional and structural changes were frequently reported in the prefrontal cortex and the basal ganglia, suggesting the role of cognitive modulation and reward processing in chronic orofacial pain. The findings highlight the potential for brain neuroimaging as an investigating tool for understanding chronic orofacial pain.

## Introduction

Trigeminal neuropathic pain (TNP) and pain related to temporomandibular disorders (TMD) have been the challenging issues in chronic orofacial pain [1], [2]. Effective management of the pain has not been established. Unlike acute orofacial pain (e.g., toothache), TNP and TMD pain may persist when the peripheral lesions have been treated, or even without peripheral abnormalities being found [3], [4]. In addition, the degree of TNP and TMD pain is associated with the cognitive and emotional factors, such as anxiety and depression [5]. Since pain is a multidimensional experience, consisting of sensory-discriminative and cognitive-affective experiences [6], TNP and TMD pain may be closely associated with the abnormality in the central pathophysiological process [7]. Therefore, the understanding of how the brain shapes pain experience would be critical to manage the pain of TNP and TMD patients.

Brain neuroimaging has been widely used to investigate the changes in brain function and structure associated with chronic orofacial pain (TNP: [8]–[17]; TMD: [10], [11], [18]–[29]). However, the conclusions drawn from a single study could be limited by its specific research conditions, including the criteria of patient selection, the experimental design and the approach of neuroimaging data analysis. And statistically, the imaging results from a single study can also be compromised by the small number of participants. The small sample size may lead to a lower statistical power, thus influencing the reproducibility of the results [30]. Therefore, in the current study we systematically reviewed the literature regarding TNP/TMD pain-related changes in brain structure (including gray matter and white matter anatomy [31]) and brain function (including the blood-oxygen-level-dependent [BOLD] signal, the cerebral blood flow and the magnetic resonance spectroscopy signal [32]). Previous investigation on the other types of chronic pain has revealed that changes in the pain-related network (including the thalamus, the primary and secondary somatosensory cortices, the mid/anterior cingulate cortex, and the insula) were associated with chronic pain [33], [34]. In the pain-related network, the alterations in the thalamocortical pathway of somatosensation are associated with pain with a central nature [35]–[37]. In addition, cognitive-affective factors, such as cognitive reappraisal, anxiety and depression, are critical to shape the experience of chronic pain [6], [38]. Based on the previous evidence, we here hypothesized that (1) both TNP and TMD would show a common pattern of functional and structural changes within the pain-related network. (2) As a central pain, TNP would show more changes in the thalamocortical pathway, compared to TMD pain, which is predominantly associated with the abnormality within the peripheral musculoskeletal system (e.g., the masticatory muscle and the joint). (3) The prefrontal cortex, the limbic system and the circuitry of reward processing (including the basal ganglia) would show functional or structural changes related to chronic orofacial pain [33], [34].

## Methods

### Procedures of Literature Search

**Figure 1:**
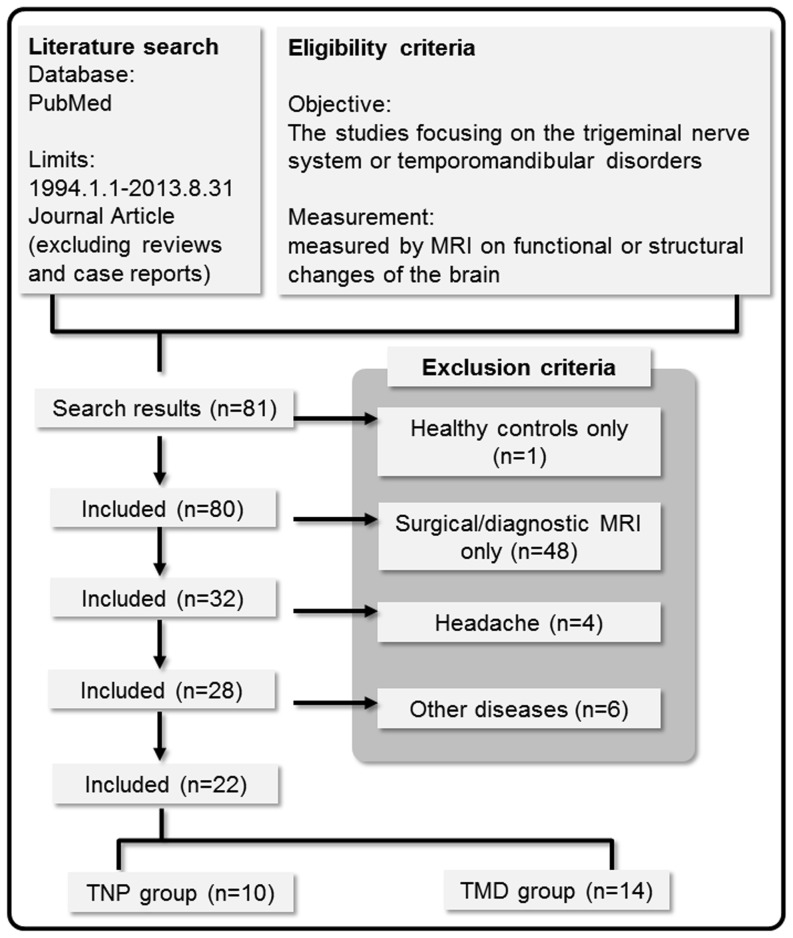
Flow diagram describing the process of review and study selection.

The current review was performed according to the guide of Preferred Reporting Items for Systematic Reviews and Meta-Analyses (PRISMA) for reporting in systematic reviews and meta-analyses [39]. To identify relevant neuroimaging studies on pain related to TNP and TMD, a computerized search of journal articles via PubMed (http://www.ncbi.nlm.nih.gov/pubmed) was conducted with several sets of keyword combinations, including “trigeminal”, “temporomandibular disorder”, “pain”, “brain” and “MRI” as the keywords (see Table 1 for the complete search strategy). We deliberately used a broad range of keyword combinations to avoid missing studies. The search was restricted to the journal articles published during the period from 1994 Jan. 1 to 2013 Aug. 31. Review articles and case reports were excluded (Figure 1).

**Table 1:** Search strategy and study selection.

Search strategy:			
**Search**	**Query**	**Filters**	**Items found**
#1	trigeminal (neuralgia OR neuropathy OR neuropathic) NOT (Case Reports[ptyp] OR Review[ptyp])	Journal Article; Publication date from 1994/01/01 to 2013/08/31	1949
#2	temporomandibular disorder NOT (Case Reports[ptyp] OR Review[ptyp])		5176
#3	pain (central nervous system OR brain) MRI		5042
#4	(#1 OR #2) AND #3		81
**Inclusion and exclusion criteria of study selection:**			
**Inclusion criteria**			
1. The studies which performed on either TNP or TMD patients.			
2. The MRI-related studies, including structural MRI (sMRI), functional (BOLD-based) MRI (fMRI), MRS, and diffusion-weighted MRI (DWI).			
**Exclusion criteria**			
1. The studies which investigated only healthy controls.			
2. The studies which applied MRI as a diagnostic tool for surgery or clinical assessment, i.e., not focusing on the long-term changes in brain signatures.			
3. The studies which focused on the trigeminal system-related disorders other than TNP and TMD, such as dental pain or headache.			

### Eligibility Criteria and Study Selection

The studies retrieved according to the above-stated search strategy (Figure 1) were further screened according to the inclusion and exclusion criteria listed in Table 1B. In this review, we only focused on the findings associated with the following five major neuroimaging approaches: (1) BOLD signal-based functional MRI (fMRI), (2) arterial spin labelling (ASL), (3) magnetic resonance spectroscopy (MRS), (4) T1-weighted structural MRI (sMRI), and (5) diffusion-weighted imaging (DWI). Note that we considered MRS signal changes here as a signature of brain function, because it reflects the metabolic changes of biochemical molecules, which underlie the transduction of neural signals. All the five imaging modalities have been widely used in investigating the brain signature of chronic diseases. For detailed introduction of the methodology about each approach, see [34], [40]–[42]. The studies that potentially used the same patient cohort with different imaging analyses were included. The eligibility assessment was performed by the first author (C-S.L.), primarily according to the title and the abstract of the selected articles.

### Data Collection and Extraction of Data Items

The full text of all the selected studies, together with the online supporting materials, was retrieved from the Internet. The following data items were manually extracted from the full text version of the original articles and classified into two categories:

1. Demographic and clinical characteristics (6 items), including the diagnosis of orofacial disorders (including subtypes), the number of participants of each sex and mean age (for both the patient and the control groups), pain severity (including the ratings of intensity and/or unpleasantness), and the duration of pain (Table 2)
2. Experimental design and neuroimaging findings (6 items), including the type of imaging modality, the type of stimuli and the site of stimulation, the covariate (e.g., pain severity) considered in imaging analysis, the signals measured by neuroimaging, and the major imaging findings (Table 3 and Table 4).

**Table 2:** Demographic and clinical profiles of the included studies.

Source	Diagnosis	Patient					Control		
		F	M	Age	Severity (0–10)	Duration (Year)	F	M	Age
**Trigeminal neuropathic pain**									
Becerra 2006	TNP	5	1	48.8^1^	>4^3^	N/A	-		
Blatow 2009	TN	14	4	48–73	N/A	N/A	8	5	25–70
		6	4	44–76	N/A	N/A			
Scrivani 2010	TNP	4	2	48.3	>3–4^3^	N/A	-		
Gustin 2011	TNP^6^	17	4	54.7	3.5/3.4^4^	8.5	24	6	53.6
Moisset 2011	TNP-i	6	9	67.2	4–6	8.3			
Gustin 2012	TNP	12	3	50	3.8/3.6^4^	4.7	27	26	41
Henderson 2013	TNP	19	4	49.8	3.9	5.8	31	12	49.8
Obermann 2013	TN^7^	36	24	62	7.7	8.3	28	21	61.8
DeSouza 2013	TN-i	15	9	48.5	N/A	6.3	15	9	47.6
DeSouza 2014	TN	11	7	54.1	N/A	N/A	11	7	49.6
**Temporomandibular joint disorder pain**									
Jiang 2006	TMD-s	6	1	26.9	N/A	1–6(mo)	5	5	31
Younger 2010	TMD-m	15	0	38	4.3^1^	4.4	15	0	N/A^5^
Abrahamsen 2010	TMD-m	18	1	40.7	4.8	12.4	-		
Nebel 2010	TMD	13	0	28.7	2.4	N/A	12	0	28.8
Zhao 2011	TMD-s	12	4	33.7	≥5	N/A	7	7	23.7
Gerstner 2011	TMD-m	9	0	25.4^1^	2.2	2.5	9	0	24.8^2^
Gustin 2011	TMD	16	4	45.7	4.7/3.2^4^	11.5	25	6	46.8
Moayedi 2011	TMD-i	17	0	33.1	4.3/5.4^2^	9.8	17	0	32.2
Weissman-Fogel 2011	TMD	17	0	35.2^1^	4.2	9.3	17	0	N/A^5^
Gerstner 2012	TMD	10	1	25.8	3.8	0.5–7	10	1	24.8
Ichesco 2012	TMD	8	0	25.4	2.2	2.5	8	0	24.9
Moayedi 2012	TMD-i	17	0	33.1	4.3/5.4^2^	9.8	17	0	32.8
Salomons 2012	TMD	17	0	33.1	4.3/5.4^2^	9.8	17	0	32.2
Gustin 2012	TMD	13	4	44	4.2/4.5^4^	10.7	27	26	41

i, idiopathic; m, myofascial; s, synovitis; F, number of female participants; M, number of male participants; N/A, not available from the full text; TNP, trigeminal neuropathic pain; TMD, temporomandibular joint disorder.

1 Mean age is calculated based on the data revealed in the original table; ^2^average pain intensity/unpleasantness over the last month; ^3^brushing-evoked/spontaneous pain; ^4^pain a week before/pain before scanning; ^5^age matched with the patient group; ^6^including TN patients; ^7^including TN patients with concomitant chronic facial pain.

**Table 3:** Experimental design and neuroimaging findings of the included studies: Trigeminal neuropathic pain.

Source	Experimental design					Major neuroimaging findings on the pain-related brain regions
	Modality	Stimuli	Site	Covariate	Signal	
Becerra 2006	fMRI	Mechanical	R V2 area	-	BOLD	AF>UF: THA/SI/R aINS/R ACC
		Thermal	(with allodynia)			
Blatow 2009	fMRI	Mechanical	R/L fingers and lips	-	BOLD	Pre-OP<HC (finger and lip): [B S1/B S2]
Scrivani 2010	fMRI	Mechanical Thermal	R/L V2 or V3 area	-	BOLD	Medication<Placebo (Thermal): R THA/R MCC/R S1
						Medication>Placebo (Mechanical): R INS/R S1
Gustin 2011	sMRI	-	-	age/sex/TBV	GMV	TNP>HC: CL pINS
						TNP<HC: B THA/IL S1/IL aINS
	fMRI	Mechanical	R bottom lips	-	BOLD	(for localizing ventroposterior THA)
	MRS	-	-	-	NAA/Cr	TNP<HC: [THA]
Moisset 2011	fMRI	Mechanical	AF/UF V2/V3 area, R hand	-	BOLD	AF>UF (evoked pain): L S1/R THA/L aINS/R ACC/L MCC
Gustin 2012	fMRI	Mechanical	IL lower lip/fingers	-	BOLD	Functional reorganization: [CL S1]
	DWI	-	-	age/sex	FA	TNP<HC: [CL S1]
	ASL	-	-	age/sex	CBF	TNP<HC: [CL S1]
Henderson 2013	sMRI	-	-	age/sex/TBV	GMV	TNP<HC: IL aINS/IL S1/B THA
	fMRI	Mechanical	lower lip	-	BOLD	(for localizing ventroposterior THA)
		Resting-state		-	FC	Negative correlation with thalamic GABA level
	ASL	-	-	age/sex	CBF	TNP<HC: [CL THA/CL S1]
	MRS	-	-		GABA	TNP<HC: [THA]
Obermann 2013	sMRI	-	-	age	GMV	TN<HC: L S1/B INS/B ACC/L THA/L S2
DeSouza 2013	sMRI	-	-	age	GMV	TN>HC: [IL THA]
				age	CT	TN>HC: [CL S1]
						TN<HC: [B ACC/IL pINS/IL aINS]
DeSouza 2014	DWI	-	-	-	FA	TN<HC: CC/cingulum/CL SLF/B pCOR

ACC, anterior cingulate cortex; AF, affected side; aINS, anterior insula; ASL, arterial spin labeling; B, bilateral; BOLD, blood-oxygen-level-dependent; CBF, cerebral blood flow; CC, corpus callosum; CL, contralateral; COR, corona radiate; CT, cortical thickness; DWI, diffusion-weighted imaging; FA, fractional anisotropy; FC, functional connectivity; fMRI, functional magnetic resonance imaging; GMV, gray matter volume; IL, ipsilateral; L, left side; MCC, mid-cingulate cortex; MRS, magnetic resonance spectrum; pINS, posterior insula; R, right side; S1, primary somatosensory cortex; S2, secondary somatosensory cortex; SC, structural connectivity (probabilistic tractography); SLF, superior longitudinal fasciculus; sMRI, structural magnetic resonance imaging; TBV, total brain volume; THA, thalamus; TNP, trigeminal neuropathic pain; UF, unaffected side; V2, the maxillary nerve; V3, the mandibular nerve; WMV, white matter volume.

**Table 4:** Experimental design and neuroimaging findings of the included studies: Temporomandibular joint disorder pain.

Source	Experimental design					Major neuroimaging findings on the pain-related brain regions
	Modality	Stimuli	Site	Covariate	Signal	
Jiang 2006	fMRI	Clenching	N/A	-	BOLD	TMD (clenching > resting): R S1/L ACC
Younger 2010	sMRI	-	-	-	GMV	TMD>HC: R aINS/B THA
						TMD<HC: R S1
Abrahamsen	fMRI	Mechanical	L V3 area	-	BOLD	Stim > No stim: R pINS/R S1
2010		Hypnosis				Hyperalgesia>Hypoalgesia: L IPL
Nebel 2010	fMRI	Mechanical	R index finger	-	BOLD	TMD>HC: B THA/CL S1/B S2/CL INS/B ACC
						TMD<HC: CL INS/CL S1/CL S2
Zhao 2011	fMRI	Clenching	R molars	-	BOLD	TMD (CL clenching>resting): B ACC
Gerstner 2011	sMRI	-	-	age	GMV	TMD<HC: L ACC/R aINS
					WMV	TMD>HC: B STG
						TMD<HC: B ACC
Gustin 2011	sMRI	-	-	age/sex/TBV	GMV	TMD v. HC: n.s.
	fMRI	Mechanical	R bottom lips	-	BOLD	(for localizing ventroposterior THA)
	MRS	-	-	-	NAA/Cr	[THA ]: TMD v. HC n.s.
Moayedi 2011	sMRI	-	-	age/TIV	GMV	[THA ]: TMD v. HC n.s.
				age	CT	TMD>HC: [R S1]
Weissman- Fogel 2011	fMRI	Stroop task		-	BOLD	TMD>HC (cognitive interference): ACC/L S1
						TMD>HC (emotional interference): L ACC
Gerstner 2012	MRS	Pressure	R anterior temporalis	-	Glu/Gln/	TMD>HC (NAA/Cho): [L pINS]
			R thumb		NAA/Cho level	
Ichesco 2012	fMRI	Pressure	L anterior temporalis	age	FC	TMD>HC: L aINS-R ACC/R aINS-R ACC
		Resting-state				TMD>HC: L aINS-R ACC/L pINS-L PHG/R aINS-R THA
Moayedi 2012	DWI	-	-	age	FA	TMD<HC: [R THA/R S1](nearby)
					SC	TMD>HC: CC-L FP
Salomons 2012	sMRI	-	-	-	CT	Correlated with helplessness- positive: [L SMA], negative: [L MCC/L PCC]
	DWI				FA	Correlated with helplessness-positive: [cingulum], negative: [CC/CST]
Gustin 2012	fMRI	Mechanical	IL lower lip/fingers	age/sex	BOLD	No functional reorganization at CL S1
	DWI	-	-	-	FA	[CL SI]: TMD v. HC n.s.
	ASL	-	-	age/sex	CBF	[CL SI]: TMD v. HC n.s.

ACC, anterior cingulate cortex; aINS, anterior insula; ASL, arterial spin labeling; B, bilateral; BOLD, blood-oxygen-level-dependent; CBF, cerebral blood flow; CC, corpus callosum; CL, contralateral; CST, cortical spinal tract; CT, cortical thickness; DWI, diffusion-weighted imaging; FA, fractional anisotropy; FC, functional connectivity; fMRI, functional magnetic resonance imaging; GMV, gray matter volume; IL, ipsilateral; L, left side; MCC, mid-cingulate cortex; MRS, magnetic resonance spectrum; pINS, posterior insula; R, right side; S1, primary somatosensory cortex; S2, secondary somatosensory cortex; SC, structural connectivity (probabilistic tractography); SLF, superior longitudinal fasciculus; sMRI, structural magnetic resonance imaging; STPI, State Trait Personality Inventory; TBV, total brain volume; THA, thalamus; TIV, total intracranial volume; TMD, temporomandibular disorder; V3, the mandibular nerve; WMV, white matter volume.

In addition, five data items related to study quality and risk of bias were extracted (see Table 5 and Table 6). In total 17 data items were extracted.

**Table 5:** Results of assessments of risk of bias and study quality: Trigeminal neuropathic pain.

Source	Criteria of patient selection		Status of medication		Standardized assessment		Statistical model		Imaging statistics		Total score
Becerra 2006	Inclusion/Exclusion^1^	1	[Y], 1-dose interval	1	QST, BDI^2^	1	FE	1	Generalized mixture model	1	5
Blatow 2009	-	0	[Y]	1	-	0	-	0	Bonferroni correction	1	2
Scrivani 2010	Inclusion/Exclusion	1	[Y], drug screening	1	QST, MADRS	1	RE^3^	1	Gaussian mixture model	1	5
Gustin 2011	Liverpool Criteria	1	[Y]	1	BDI, STAI, MPQ	1	RE	1	FDR	1	5
Moisset 2011	Inclusion/Exclusion	1	[Y], 12 hrs	1	QST, NPSI	1	RE	1	uncorrected	0	4
Gustin 2012	Liverpool Criteria	1	[Y]	1	MPQ	1	RE	1	FWE	1	5
Henderson 2013	Liverpool Criteria	1	[Y]	1	MPQ	1	RE	1	FDR	1	5
Obermann 2013	Inclusion/Exclusion	1	[Y]	1	-	0	-	0	FWE	1	3
DeSouza 2013	Inclusion/Exclusion	1	[Y]	1	-	0	Permutation testing	1	corrected based on permutation testing	1	4
DeSouza 2014	Inclusion/Exclusion	1	[Y]	1	-	0	Permutation testing	1	TFCE	1	4

BDI, Beck Depression Inventory; BPI, Brief Pain Inventory; FDR: control for false discovery rate; FWE: control for family-wise error; MADRS, Montgomery-Asberg Depression Rating Scale; (s)MPQ, (short-form) McGill Pain Questionnaire; NPSI, neuropathic pain symptom inventory; STAI: State-Trait Anxiety Inventory; TFCE; Threshold-Free Cluster Enhancement [85].

1 Details about the criteria were not found; ^2^The use of BDI was only noted in Figure S1; ^3^using FMRIB's local analysis of mixed effects; [Y] denotes that the status of medication was reported.

**Table 6:** Results of assessments of risk of bias and study quality: Temporomandibular joint disorder pain.

Source	Criteria of patient selection		Status of medication		Standardized assessment		Statistical model		Imaging statistics		Total score
Jiang 2006	-	0	-	0	-	0	RE	1	uncorrected	0	1
Younger 2010	Inclusion/Exclusion criteria	1	[Y]	1	-	0	-	0	FDR	1	3
Abrahamsen 2010	RDC	1	-	0	SCL, HGSH:A	1	RE	1	FWE	1	4
Nebel 2010	RDC	1	-	0	sMPQ	1	RE^2^	1	Cluster-corrected	1	4
Zhao 2011	CDC	1	-	0	SCL-90	1	-	0	-	0	2
Gerstner 2011	RDC	1	3 days^3^	1	sMPQ, BPI, STPI	1	Permutation testing	1	corrected based on permutation testing	1	5
Gustin 2011	RDC	1	[Y]	1	BDI, STAI, MPQ	1	RE	1	FDR	1	5
Moayedi 2011	RDC	1	[Y], 24 hrs	1	NEO-FFI	1	-	0	FDR, Bonferroni correction	1	4
Weissman- Fogel 2011	RDC	1	[Y], 24 hrs	1	-	0	-	0	FDR	1	3
Gerstner 2012	RDC	1	2 wks	1	sMPQ, STPI	1	RE	1	-^3^	0	4
Ichesco 2012	RDC	1	3 days^1^	1	sMPQ, BPI, STPI	1	RE	1	Cluster-corrected	1	5
Moayedi 2012	Inclusion/Exclusion criteria	1	-	0	-	0	Permutation testing	1	TFCE or cluster-mass corrected	1	3
Salomons 2012	Inclusion/Exclusion criteria	1	[Y], 24 hrs	1	PCS	1	-	0	FWE	1	4
Gustin 2012	RDC	1	[Y]	1	MPQ	1	RE	1	FWE	1	5

BDI, Beck Depression Inventory; BPI, Brief Pain Inventory; CDC, Clinical Diagnostic Criteria [84]; FDR: control for false discovery rate; FWE: control for family-wise error; HGSHS:A, Harvard Group Scale of Hypnotic Susceptibility Form A; (s)MPQ, (short-form) McGill Pain Questionnaire; PCS: Pain Catastrophizing scale; RDC: Research Diagnostic Criteria [44]; SCL, Symptom Check List; STAI: State-Trait Anxiety Inventory; STPI, State-Trait Personality Inventory; TFCE; Threshold-Free Cluster Enhancement [85].

1 Nonsteroidal anti-inflammatory drugs; ^2^using FMRIB's local analysis of mixed effects; ^3^the study directly compared the metabolite level resulted from the selected ROIs. [Y] denotes that the status of medication was reported.

### Assessment of Study Quality and Risk of Bias

In the current PRISMA guideline, the assessment of risk of bias and study quality has been clearly distinguished [39]. For the assessment of risk of bias, we considered the heterogeneity of diagnosis and symptomatology as the major factor that would introduce bias in research outcomes. Therefore we focused on the inclusion/exclusion criteria for patient selection, the patients' history of medication and the use of standardized clinical assessments (i.e., the items 1–3). For the assessment of study quality, we applied the recently proposed guideline on reporting functional MRI studies [43], focusing on the statistical model for comparison and imaging statistics (i.e., the items 4–5):

1. Criteria of patient selection: whether established criteria (e.g., the Research Diagnostic Criteria [RDC], [44]) or detailed inclusion/exclusion criteria were applied for patient selection.
2. Statuts of medication: whether the history of prescription has been recorded and the medication was temporally discontinued before the MRI scan.
3. Standardized assessment: whether standardized pain/psychological assessments were applied to assess the patients.
4. Statistical model: whether the type of group-wise statistical inference (e.g., random or fixed effect) was reported.
5. Imaging statistics: whether correction of multiple comparison was applied to the resulted images.

Each item was scored as 1 if the related details were reported in the study, otherwise as 0. The total score of a study ranged from 0 to 5.

### Qualitative Meta-Analysis

In the current review, we did not perform a quantitative imaging meta-analysis because of the heterogeneity of imaging modalities and analysis approaches, and the small number of participants in some studies. Instead, we performed a qualitative meta-analysis by identifying the brain regions that showed significant functional or structural changes in patients, compared to the healthy control participants, or within the patients between the affected and the unaffected sites. The reported brain regions were categorized into 14 regions of interest (ROIs) that are associated with pain processing [33]: the thalamus, the primary and secondary somatosensory cortices (S1/S2), the anterior and mid-cingulate cortices (ACC/MCC), the anterior and posterior insular cortices (aINS/pINS), the superior, middle, inferior and orbitofrontal part of the prefrontal cortex (PFC), the basal ganglia (BG), the medial temporal lobe (including the hippocampus and the amygdala, MT), and the periaqueductal gray (PAG). Categorization was performed separately for the positive changes (e.g., increased BOLD activity or increased GMV in patients vs. healthy control participants) and the negative changes (e.g., decreased BOLD activity or decreased GMV in patients vs. healthy control participants).

The anatomical labels of the ROIs were surveyed using FSLView (http://fsl.fmrib.ox.ac.uk/fsl/fslview/). The Oxford thalamic connectivity atlas [45] was consulted for specifying the functional role of the thalamic sub-regions, according to their patterns of cortical connections. The Jülich histological atlas (http://www.fz-juelich.de/inb/inb-3/Home) was consulted for specifying the Brodmann area of the S1.

### Statistical Analysis

#### Quantifying the similarity and distinctiveness of brain signature pattern

Our main hypothesis focused on the similarity and distinctiveness of the pattern of brain signature related to TNP and TMD pain. We quantified the *brain signature pattern* based on the changes in the pain-related ROIs, according to the following definitions:

- For each ROI, we defined the number of the studies that reported significant changes, as shown in the qualitative meta-analysis, as the index *regional change* of that ROI. This index was calculated respectively for positive and negative changes and for different imaging modalities. For example, in the functional studies about TNP and TMD, the positive regional change for S1 was ‘3’ and ‘4’, respectively (Table 7).
- For the positive and negative changes of different imaging modalities, the brain signature pattern of the TNP or the TMD group was represented as a set of regional changes from the nine ROIs: THA, S1, S2, PFC, BG, MT, PAG, the cingulate cortex (CC, including ACC and MCC), and the insula (INS, including aINS and pINS).

**Table 7:** The findings of meta-analysis on functional changes by pain-related regions: BOLD/CBF.

Source	Positive changes									Negative changes								
	THA	S1	S2	CC	INS	PFC	BG	MT	PAG	THA	S1	S2	CC	INS	PFC	BG	MT	PAG
**Trigeminal neuropathic pain**																		
Becerra 2006^1^	PM	2		a	a	mi	↑			↓					↓	↓	↓	
Blatow 2009											*	*						
Scrivani 2010^2^	PF	1		m		smio	↑				↓			a	i			
Moisset 2011^1^	PM	3b		am	a	i	↑	↑		N/A	N/A	N/A	N/A	N/A	N/A	N/A	N/A	N/A
Gustin 2012^3^											*							
Henderson 2013^3^										*	*							
**Temporomandibular joint disorder pain**																		
Jiang 2006		2		a		o	↑			N/A	N/A	N/A	N/A	N/A	N/A	N/A	N/A	N/A
Abrahamsen 2010		2			p	m				N/A	N/A	N/A	N/A	N/A	N/A	N/A	N/A	N/A
Nebel 2010	PF	1/3b		m	p			↑			2/1	↓		p				
Zhao 2011				a		mi				N/A	N/A	N/A	N/A	N/A	N/A	N/A	N/A	N/A
Weissman-Fogel 2011^4^		1		a		sm	↑	↑		N/A	N/A	N/A	N/A	N/A	N/A	N/A	N/A	N/A
Ichesco 2012^5^	PF			a		s		↑		N/A	N/A	N/A	N/A	N/A	N/A	N/A	N/A	N/A

See Table 3 for the abbreviations of the brain regions. N/A: The study did not report the findings of negative changes. The asterisk denotes that the finding was derived from a ROI-specific analysis. Upward and downward arrows denote positive and negative changes, respectively (without showing the sub-region of the change). a, anterior; m, mid; p, posterior; i, inferior; s, superior; o, oribitofrontal; PM, connection with the premotor cortex; PF, connection with the prefrontal cortex.

1 Affected side > Unaffected side; ^2^the findings shown in ‘Decreased activation’ represents ‘placebo>drug’; ^3^baseline cerebral blood flow; ^4^effect of cognitive and emotional interference; ^5^increased resting-stated/pain-evoking functional connectivity between the insula and these regions.

Notably, we here focused on the studies that applied whole-brain analysis. The findings derived from ROI-specific analyses were excluded (e.g., [9]). It should be clarified that an analysis was here considered as ‘whole-brain’, if a greater mask was applied to restrict the search in the cortical area (e.g., [17]). The findings from the MRS studies ([10]–[12]) were also excluded, because all the studies applied ROI-specific analyses. The analyses were performed separately for functional and structural studies and for the reports of positive and negative changes. Because there were very few findings from the functional studies with negative changes and the structural studies with positive changes (for descriptive data see Figure S1), we focused only on (1) the functional studies with positive changes and (2) the structural studies with negative changes. Similarity or distinctiveness of the brain signature patterns related to TNP and TMD was judged by the following two conditions: (1) there is a significant association in the regional changes between the two groups, and (2) there is no significant difference in the regional changes between the two groups.

#### Analysis on the association of regional changes

We considered the brain signature patterns related to TNP and TMD as similar, if the brain signature changes of one group were associated with those in another group. We performed the non-parametric Spearman rank correlation analysis on the regional changes, between TNP and TMD. A significantly positive correlation indicated that the brain signature pattern between the two groups was associated. The correlation coefficient with a p value <0.05 was considered statistically significant.

#### Analysis on the overall difference of regional changes

To claim that the patterns are similar between the two groups, we considered that the overall degree of regional changes (e.g., the mean regional changes over the nine ROIs) not to be significantly different. It should be noted that a direct comparison in regional change could be biased by the total number of articles related to TNP and TMD. Therefore, we compared the *normalized regional change*, i.e., the original index (i.e., regional change) divided by the total number of articles. We performed the two-tailed paired Wilcoxon test on the normalized regional changes between TNP and TMD. The resulting W statistics quantifies the difference in the overall brain signatures pattern between the two groups. Note that the null hypothesis referred to ‘no difference between the two groups’. The W statistics with a p value <0.05 was considered statistically significant.

#### Decision-making on the similarity and distinctiveness of brain signature

The pattern of brain signature between TNP and TMD was categorized as ‘common’ if the Spearman rank correlation coefficient was significant AND the paired Wilcoxon test failed to reject the null hypothesis. In contrast, it was categorized as ‘different’ if the Spearman rank correlation coefficient was not significant AND the paired Wilcoxon test rejected the null hypothesis.

## Results

### Included Studies

According to the strategy of literature search (Table 1), in total 81 studies were included as the original set of studies (Figure 1). Among this set, 59 studies were excluded according to the pre-defined exclusion criteria (Table 1B): (1) one study investigating only the healthy control participants; (2) 48 studies applying MRI as a diagnostic tool (e.g., for surgical assessment [46] or examination of vascular pathology [47]); (3) 10 studies focusing on the disorders other than TNP and TMD, including 4 studies of headache (mostly about migraine) and 6 studies of other disorders (Table 1B). In total, 22 studies were included as the final set for qualitative meta-analysis (Figure 1). Full text was retrieved from all the studies. Twenty studies were originally published in English and two in Chinese ([20], [27]). Within the final set (n = 22), 8 studies exclusively recruited TNP patients; 12 studies exclusively recruited TMD pain patients, and two studies recruited both TNP and TMD pain patients ([10], [11]). In total 10 TNP-related and 14 TMD pain-related studies were included.

### Demographic and Clinical Profiles

#### Diagnosis

The studies under review diverted in the diagnostic categories (Table 2). In the TNP studies, 4 out of 10 studies focused exclusively on trigeminal neuralgia (TN), an episodic form of neuropathic pain [48]. The other 6 studies included TNP with a variety of etiologies (e.g., trauma or post-herpetic infection, [8]). The heterogeneity of diagnosis was also found in the TMD studies. Three out of 14 TMD studies focused exclusively on the myofascial-type TMD ([18], [26], [28]), two on idiopathic (non-traumatic) TMD ([21], [22]), and 2 on inflammatory-type TMD (synovitis, [20], [27]).

#### The demographic profiles of the patients

The sample size of the patient group was relatively small (n<12) in two TNP studies [8], [14] and in 4 TMD studies [19], [20], [28], [29] (Table 2). There were more females patients recruited than male patients (Table 2). In the TNP studies, the ratio of female to male patients was approximately 2∶1. In the TMD studies, the number of female patients was even overwhelmingly higher than the number of the male patients, with eight studies exclusively recruiting only female patients ([13], [19], [22]–[26], [28]). The age range of the patients in the TNP and the TMD studies was approximately 45–55 y/o and 25–45 y/o, respectively (Table 2). The patients in the TNP studies were, in general, older than the patients in the TMD studies. One study has patients with a mean age >65 y/o ([13]), which were considered the older population. The sex and age profiles of the patients, in general, were consistent with the previous epidemiological findings of the disorders [48].

#### The clinical profiles of the patients

Most studies set minimal severity of pain as the inclusion criteria, such as a score greater than 4 on a 0–10 visual analogue scale (VAS). The definition of pain severity, however, varied across studies. While some studies defined it as the current pain intensity (e.g., [18], [28]), other studies defined it as the average pain over a month (e.g., [21], [22], [24]) or the pain a week before scanning (e.g., [10], [11]). In general, both the TNP and TMD studies showed pain severity at the moderate level (mean pain intensity <5 on a 0–10 VAS, Table 2). In the TMD studies, the patients with mild pain were included in two studies ([19], [23]). The duration of pain, on contrary, varied significantly between studies and between patients (Table 2). In the TNP studies, the mean duration of pain ranged from 4.7 to 8.5 years, across 6 studies. In the TMD studies, the mean duration of pain ranged from 2.5 to 12.4 years, across 11 studies.

### Experimental Design

#### Imaging modalities

The TNP studies included 6 investigations on the structural changes (sMRI/DWI = 4/2) and 9 investigations on the functional changes (fMRI/ASL/MRS = 5/2/2). The TMD studies included 8 investigations on the structural changes (sMRI/DWI = 5/3) and 10 investigations on the functional changes (fMRI/ASL/MRS = 7/1/2) (Table 4). In two studies, the fMRI investigations were performed mainly for functionally localizing a specific ROI ([11], [12]). These investigations were not included in the subsequent meta-analysis.

#### Stimulation models and tasks

In the TNP studies, 7 studies have applied mechanical stimuli (brushing) to the subjects ([8]–[14]) (Table 3). Two studies additionally applied thermal (cold and heat) stimuli ([8], [14]) to evoke pain or allodynia. In the TMD studies, 4 studies applied mechanical stimuli to the subjects ([10], [11], [18], [23]) and one study applied pressure stimuli ([19]) to evoke pain. Two studies used a clenching task to evoke pain [20], [27]. Behavioral tasks were applied in two studies: one study applied hypnotic modulation to modulate pain experience ([18]) and another study applied the Stroop task to assess the attentional interference related to pain ([25]).

### Assessment of Study Quality and Risk of Bias

In general, the included 22 studies showed a moderate to high score of study quality and risk of bias assessment. The score (mean±standard deviation) of the TNP studies is 4.2±1.0 and that of the TMD studies is 3.7±1.2. There was no significant difference in the assessment score between the two groups (two-tailed independent t-test *p* = 0.3).

#### Criteria of patient selection

Twenty-two studies have reported the detailed criteria about patient selection (TNP: 9/10; TMD: 13/14). In the TMD studies, the Research Diagnostic Criteria (RDC) [44] was the most frequently adopted diagnostic criteria [11], [18], [19], [21], [23], [25], [28], [29].

#### Status of medication

Nineteen studies have reported the status of medication of the patients (TNP: 10/10; TMD: 9/14). However, only 9 studies have reported that the medications were temporally discontinued before MRI scanning. Among these studies, the duration of medications discontinuation varied from 2 weeks to only one drug interval.

#### Standardized assessment

Standardized assessments of pain, such as the McGill Pain Questionnaire [49], [50] or Quantitative Sensory Testing (QST) [51], were performed in some of the studies (TNP: 6/10; TMD: 6/14). The psychological assessments regarding the chronic pain-related moods, such as the Beck Depression Inventory (BDI, [52]) for depression and the State-Trait Anxiety Inventory (STAI) for anxiety, were not widely used.

#### Statistical model

Twelve studies applied the random-effect (RE) model for the group-wise comparison. With this model, a conclusion drawn from the imaging results can be inferred to the population [53]. The fixed-effect (FE) model was suggested for a study with a small sample size [54]; however, it was only applied in one of the small-sample-size studies [8]. The permutation testing [55], a non-parametric approach for imaging analysis, was applied in four studies [16], [17], [22], [28].

#### Imaging statistics

Twenty studies have applied some form of correction of multiple comparison in the imaging results (TNP: 9/10; TMD: 11/14). This item of assessment was not applied to one study [29], which only reported findings from an ROI-based but not a whole-brain analysis.

### Findings from the Qualitative Meta-analysis

#### Changes in brain function

The results on brain function included the investigations on BOLD/CBF and MRS studies (Table 7 and Table 8). For both TNP and TMD patients, the S1, the ACC/MCC and the PFC are the most consistently reported regions (S1: 7 studies; ACC/MCC: 8 studies; PFC: 8 studies) (Table 7, Table 8 and Figure S1), while the S2 activation was only reported in 2 studies (Table 7). These findings from BOLD/CBF/MRS changes have revealed predominantly positive changes. The pattern of changes is more consistent in the TNP studies, where three studies have consistently reported an increased BOLD activation at the thalamus, S1, ACC/MCC and PFC [8], [13], [14]. In contrast, the pattern in the TMD group was less consistent across studies. Notably, concurrent increased/decreased activation was found between the thalamus and the S1 [8], [12]–[14], [23], and between the PFC and the basal ganglia [8], [13], [14], [20], [25]. Within the PFC, the superior or middle frontal gyrus (i.e., the dorsolateral PFC) was more frequently reported than the medial or orbital part of the PFC.

**Table 8:** The findings of meta-analysis on functional changes by pain-related regions: MRS.

Source	Positive changes									Negative changes								
	THA	S1	S2	CC	INS	PFC	BG	MT	PAG	THA	S1	S2	CC	INS	PFC	BG	MT	PAG
**Trigeminal neuropathic pain**																		
Gustin 2011^1^										*								
Henderson 2013^2^										*								
**Temporomandibular joint disorder pain**																		
Gerstner 2012^3^					p*													

See Table 3 for the abbreviations of the brain regions. The asterisk denotes that the finding was derived from a ROI-specific analysis. p, posterior.

1 NAA/Cr level; ^2^GABA level; ^3^NAA/Cho level.

For the negative changes, a decreased NAA/Cr/GABA level was found in the thalamus only in the TNP but not the TMD patients [11], [12]. In contrast, a decreased NAA/Cho level was found in the TMD patients [29] (Table 8). The TNP patients also showed decreased baseline CBF in the thalamus and the S1, which were not reported in the TMD patients [10], [12].

#### Changes in brain structure

The results on brain structure included the investigations on gray matter and white matter (Table 9 and Table 10). For both TNP and TMD studies, there were more findings on negative changes than positive changes (Figure S1I). Again, the brain regions with significant changes were predominantly the pain-related regions, including the thalamus, S1, ACC/MCC, and the insula. Concurrent changes were found between the thalamus and the S1 [11], [15], [17], [22], and additionally between the ACC/MCC and the insula [15], [17], [28]. Changes on white matter were exclusively negative changes. Three studies have consistently reported decreased FA nearby the S1 [10], [16], [22] (Table 10).

**Table 9:** The findings of meta-analysis on structural changes by pain-related regions: Gray matter.

Source	Positive changes									Negative changes								
	THA	S1	S2	CC	INS	PFC	BG	MT	PAG	THA	S1	S2	CC	INS	PFC	BG	MT	PAG
**Trigeminal neuropathic pain**																		
Gustin 2011					p					PF	3b			a		↓		
Obermann 2013										TP	1	↓	a	a	dlo	↓		
DeSouza 2013^1^	PF^2^	2/1				pl	↑	↑	↑				a	p	o			
**Temporomandibular joint disorder pain**																		
Younger 2010	PF				a	i	↑				3b							
Gerstner 2011													m	a	i		↓	
Moayedi 2011^1^		3b*				vl*												

See Table 3 for the abbreviations of the brain regions. The asterisk denotes that the finding was derived from a ROI-specific analysis. Upward and downward arrows denote positive and negative changes, respectively (without showing the sub-region of the change). a, anterior; m, mid; p, posterior; i, inferior; oribitofrontal; vl, ventrolateral; dl, dorsolateral; PF, connection with the prefrontal cortex; TP, connection with the temporal cortex.

1 cortical thickness; ^2^gray matter volume.

**Table 10:** The findings of meta-analysis on structural changes by pain-related regions: White matter.

Source	Positive changes									Negative changes								
	THA	S1	S2	CC	INS	PFC	BG	MT	PAG	THA	S1	S2	CC	INS	PFC	BG	MT	PAG
**Trigeminal neuropathic pain**																		
Gustin 2012											↓							
DeSouza 2014^1^											↓	↓	m		↓			
**Temporomandibular joint disorder pain**																		
Gerstner 2011^2^													a		smi			
Moayedi 2012										*	*							

See Table 3 for the abbreviations of the brain regions. The asterisk denotes that the finding was derived from a ROI-specific analysis. Downward arrows denote negative changes (without showing the sub-region of the change). a, anterior; m, mid; i, inferior; s, superior.

1 The brain regions affected by the white matter tracts, inferred from the Discussion; ^2^white matter volume.

### Statistical Analysis

**Figure 2:**
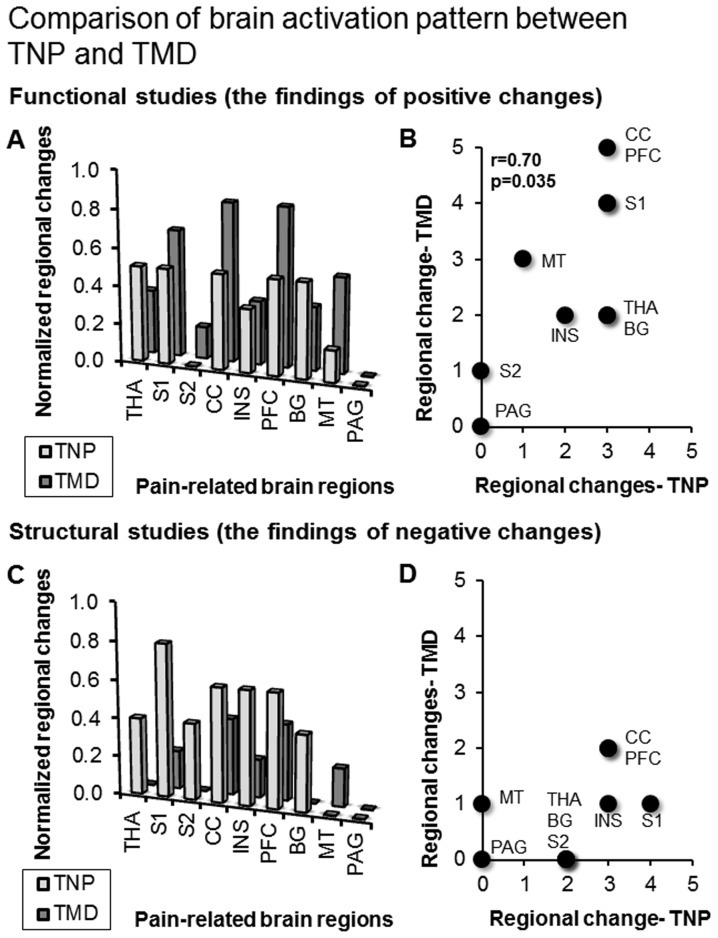
Results of the statistical analysis. Panel (A) and (B) show the pattern of brain signature from the functional studies with positive changes. Between TNP and TMD, the overall pattern did not significantly differ (A), and the pattern across each ROI was significantly correlated (B). Panel (C) and (D) show the pattern of brain signature from the structural studies with negative changes. Between TNP and TMD, the overall pattern significantly differed (C), and the pattern across each ROI was not significantly correlated (D). See Table 3 for the abbreviations.

The correlation analysis and the paired Wilcoxon test were performed on the regional changes of the pain-related ROIs between the TNP and the TMD groups, separately for the functional studies with positive changes and the structural studies with negative changes. In the functional studies with positive changes, the paired Wilcoxon test failed to reject the null hypothesis (W = 1.56, p = 0.12), and the correlation analysis showed a significant correlation (Spearman's rho = 0.70, p = 0.035) (Figure 2A–B). In contrast, in the structural studies with negative changes, the paired Wilcoxon test rejected the null hypothesis (W = 2.28, p = 0.023), and the correlation analysis did not show a significant correlation (Spearman's rho = 0.61, p = 0.083) (Figure 2C–D). Therefore, according to our criteria, the regional changes from the functional studies with positive changes showed similar brain signature pattern between TND and TMD, and the regional changes from the structural studies with negative changes showed distinct brain signature pattern between TND and TMD.

## Discussion

### Common Brain Signature related to TNP and TMD pain

We hypothesized that the two chronic orofacial pain disorders are associated with a common pattern of brain signature. This hypothesis was partially supported. The statistical analysis revealed similar pattern of brain signature only in the functional studies with positive changes (Table 7–10 and Figure 2A–B). In contrast, the structural studies with negative changes showed distinct pattern (Table 7–10 and Figure 2C–D). Among the major pain-related ROIs, changes at the thalamus, the S1, the ACC/MCC, and the insula were frequently reported. In contrast, changes at the S2 were relatively fewer. The significance of each of the regions was discussed as follows.

#### The thalamus and the S1

Both the thalamus and the S1 are critical regions of the trigeminal nerve system [56] and play a major role in the thalamocortical pathway [12]. In line with these findings, we found that the thalamus and S1 were the most frequently reported brain regions in functional and structural investigations. Notably, the thalamus and the S1 showed a higher frequency of concurrent changes. In the 11 studies that showed functional or structural changes in the thalamus, 8 studies showed concurrent changes in the S1 (positive changes: [8], [13], [14], [17], [23]; negative changes: [11], [12], [15]). The findings highlight the role of the thalamocortical pathway in chronic pain [12], [34].

#### The S2

Compared to the other pain-related brain regions, the S2 showed a lesser degree of changes in both brain function and structure. The S2, together with the insula and the mid-cingulate cortex, is considered as the key brain region evoked by noxious stimuli [57]. Its activation reliably predicted the experience of acute pain [58], [59]; however, its role in chronic pain is more elusive[33]. Animal studies have shown that both the S1 and S2 show reorganization after peripheral lesioning [60]. Human fMRI findings revealed that changes in individual perceptual threshold is linearly correlated with the degree of re-organization of the S1, but not the S2 [61]. Therefore, the S2 changes may predominantly reflect a transient change of pain experience (e.g., acute evoked pain), rather than a long-term alteration in pain processing.

#### The ACC/MCC and the insula

Both the ACC/MCC and the insular activation is frequently associated with noxious stimuli and encodes pain experience [57], [59]. The ACC activation was associated with the magnitude of subjective pain, while the MCC activation was associated with the magnitude of stimuli intensity [34], [62]. In terms of brain connectivity, the ACC and the insula are the major regions of the salience network [63]. Recent evidence has revealed that altered activity of the salience network is associated with the endogenous and self-sustaining nature of chronic pain [64]. In migraine patients, the salience network showed aberrant intrinsic connectivity [65]. In terms of brain structure, the pain sensitizers showed significant reduction in gray matter density in the ACC and the insula [66]. In line with the findings, in the patients with chronic orofacial pain, we found predominantly positive changes in brain function and negative changes in brain structure in the regions. The finding may suggest an altered subjective experience of pain in the chronic orofacial pain patients.

### Distinct Brain Signature related to TNP and TMD pain

In contrast to the similarity of activation pattern from the functional studies with positive changes, in the structural studies with negative changes, we found distinct brain signature, which may be specific to the TNP and TMD patients. Firstly, the TMD patients showed overall fewer negative changes in brain structure than the TNP patients (Figure 2C). In the TNP patients, the negative changes may indicate a loss-of-inhibition mechanism in modulating the thalamocortical pathway [12]. In addition, there were more concurrent changes in brain function between the thalamus and the S1 in the TNP studies (Table 7–10), suggesting that in the TNP patients, pain are more associated with the thalamocortical pathway, compared to the TMD patients. A recent meta-analysis has shown that, in the patients with neuropathic pain, the thalamus showed hypoactivity during resting but hyperactivity during allodynia [67]. The resting hypoactivity may be associated with sensory deafferentation related to neuropathy [67]. Our findings supported the proposal by showing decreased baseline activity (including CBF and MRS signals) and increased stimuli-evoked activity at the thalamus and the S1 ([10]–[12], see Table 7 and Table 8).

The functional studies showed that activation at the aINS was seen mainly in the TNP group, while activation at the pINS was seen in the TMD group (Table 7). It has been widely established that the anterior and the posterior sub-division of the insula play a different role in pain processing. The aINS and the pINS, respectively, showed a greater degree of functional and structural connection with the cognitive-affective network (e.g., the PFC) and the sensory discriminative network related to pain (e.g., the S1 and S2) [68], [69]. The segregation could be explained based on each etiology and symptomatology. In TNP patients, pain would like to emerge spontaneous and continuously, and therefore the aINS would play a major role in shaping such a salient experience. By contrast, in TMD patients, pain is more likely to be triggered from the peripheral tissue, and therefore a sensory-discriminative network, including the pINS, would be involved.

It should be noted that both the patterns of insula and thalamus connectivity were indirectly concluded by relatively small samples. Still, the findings strengthen the general hypothesis that plasticity in connectional pattern, rather than regional activity, would account for the difference in pain experience [70], [71]. The findings therefore highlight the importance for investigation the chronic orofacial pain at the level of brain connectome.

### The Role of Cognitive-Affective Network

Supporting our third hypothesis, we found profound functional and structural changes in the PFC, including the dorsolateral PFC (the superior or middle frontal gyrus), the ventrolateral PFC (the inferior frontal gyrus) and the orbitofrontal cortex (Table 7 and Figure 2). The orbitofrontal cortex plays a critical role in coping with pain and shows deficit activation in the neuropathic pain patients [67]. The dorsolateral and ventrolateral PFC play a key role in modulating pain, particularly via cognitive re-appraisal [6]. The consistent engagement of the PFC in both TNP and TMD pain highlights the role of psychological factors in chronic orofacial pain [38].

Notably, we found concurrent functional and structural changes between the PFC and the basal ganglia, and partly with the limbic system. The basal ganglia are the neural substrates critical for motivation and reward learning [72], and the basal ganglia-cortical loop is associated with the prediction of future reward [73]. The experience of pain relief, as a reward, is particularly important to chronic pain patients. The changes of the basal ganglia-PFC pathways may be associated with the transition from acute to chronic pain [34].

### Further Considerations and Clinical Implications

The current review showed a great variation in the criteria of patient selection and the study designs across the studies (Table 5 and Table 6). Either TNP or TMD can be categorized into different sub-types, which differ in etiology and symptomatology. For example, trigeminal neuralgia is associated with an episodic shooting and sharp pain, while the painful trigeminal neuropathy is associated with a continuous dull or sharp pain [48]. Therefore, it is crucial to systematically investigate the signs and symptoms presented by the patient group. For example, the thalamic hypoactivity may be associated with sensory deafferentation [67], and QST would be a critical tool for investigating the thalamic changes associated with the neuropathic pain. In addition, the changes in brain function and structure can be influenced by a variety of factors, such as age [74]–[76] and chronic distress [77]. Therefore, to sharpen the association between symptoms and brain changes, it would be important to clarify the effect from these confounding factors.

A general theme underlying the disease-related alterations in brain signature is neuroplasticity, which refers to the changes in brain organization that account for various forms of behavioral modifiability [78]. Brain neuroplasticity related to pain may represent the somatic memories of pain, that are sculpted by injury (e.g., peripheral noxious stimuli) or experience (e.g., pain-related distress) [79]. Therefore, investigations on neuroplasticity of brain function and structure may help explain how the experience of chronic orofacial pain is shaped. Plasticity referred to ‘an intrinsic property of the nervous system retained throughout a lifespan’, which can be molded by environmental changes and experiences [80]. In contrast, the term *neuroelasticity* referred to an adaptive process of plastic effect, in which functional or structural plasticity of the brain occurs dynamically in accordance with the addition or removal of stimuli [81]. From the clinical perspective, the concept of adaptive neuroelasticity could be useful, because it regards the brain reorganization as a dynamic course, which corresponds to the exacerbation or relief of the illness status. For example, in patients with chronic low back pain, the pain-related decrease in cortical thickness was reversed after surgical treatment [82]. In the patients with painful osteoarthritis, the pain-related decrease in GMV was reversed to the level observed in healthy controls, after successful surgery [81], [83]. The neural mechanisms of such elastic and adaptive changes in brain organization are still unclear. However, these recent findings suggest that the plastic changes in brain functions and structure, as a brain marker, would help the clinicians not only in prognosis but also in assessing the effect of the treatment of chronic orofacial pain.

### Conclusions

The current review has revealed that TNP and TMD patients showed a common pattern of brain signature regarding changes in brain function. In contrast, the pattern of structural changes differed from each other. The alterations in the thalamocortical pathway differed between the TNP and the TMD pain patients. In addition, changes in the PFC and the basal ganglia suggested the role of cognitive modulation and reward processing in chronic orofacial pain. The findings highlight the potential for brain neuroimaging as an investigating tool for understanding chronic orofacial pain.

## Supporting Information

Figure S1 **Results of qualitative meta-analysis.** The charts revealed the number of studies that showed significant changes in each of the pain-related regions. Figure S1A–C showed both functional and structural changes; Figure S1D–F showed functional changes, and Figure S1G–I showed structural changes, in trigeminal neuropathic pain (TNP), temporomandibular joint disorder (TMD) pain, and both disorders (TNP+TMD).
(TIF)

Checklist S1 **PRISMA checklist.**
(DOC)
